# Hashimoto’s thyroiditis masquerading as acute tubular injury and rhabdomyolysis

**DOI:** 10.1590/2175-8239-JBN-2024-0022en

**Published:** 2024-08-09

**Authors:** Gerry George Mathew, Varadharajan Jayaprakash

**Affiliations:** 1 SRM Medical College Hospital and Research Centre, Department of Nephrology, Chengalpattu, India.

**Keywords:** Hashimoto Disease, Acute Kidney Tubular Necrosis, Rhabdomyolysis

## Abstract

Hashimoto’s thyroiditis manifesting as hypothyroidism has been implicated in glomerular disorders due to autoantibody formation. Here we present the case of a 26-year-old male without any comorbidities presenting with easy fatiguability and weight gain for 2 months. He was found to have a creatinine of 2.1 mg/dL with a history of rhinitis treated with anti-histaminic three days prior to the hospital visit. He had symptoms of intermittent myalgia for the past two weeks. On laboratory evaluation, he was found to have raised CPK, elevated TSH, low normal T4, and positive anti-TPO and anti-Tg antibodies. Neck ultrasound revealed linear echogenic septations in the thyroid gland. Renal biopsy revealed acute tubular injury. Appropriate thyroxine supplementation was started and his creatinine decreased to 1.2 mg/dL after 1 month. It is important that clinicians should be aware of this rare kidney presentation in Hashimoto’s thyroiditis.

## Introduction

Thyroid hormones are vital players in structural and functional integrity of kidney and skeletal muscles^1,2^. Hashimoto’s thyroiditis usually manifests as hypothyroidism due to destruction of thyrocytes with presence of auto antibodies and has characteristic ultrasound features^3,4^. Rhabdomyolysis has been sparsely documented in severe hypothyroidism in medical literature^5,6^. Autoimmune thyroiditis has been implicated in various glomerular pathologies due to the phenomenon of auto-antibody formation^7^. Here we report a rare kidney manifestation of autoimmune thyroiditis, which has an important implication in terms of diagnosis and management.

## Case Description

**Figure 1:**
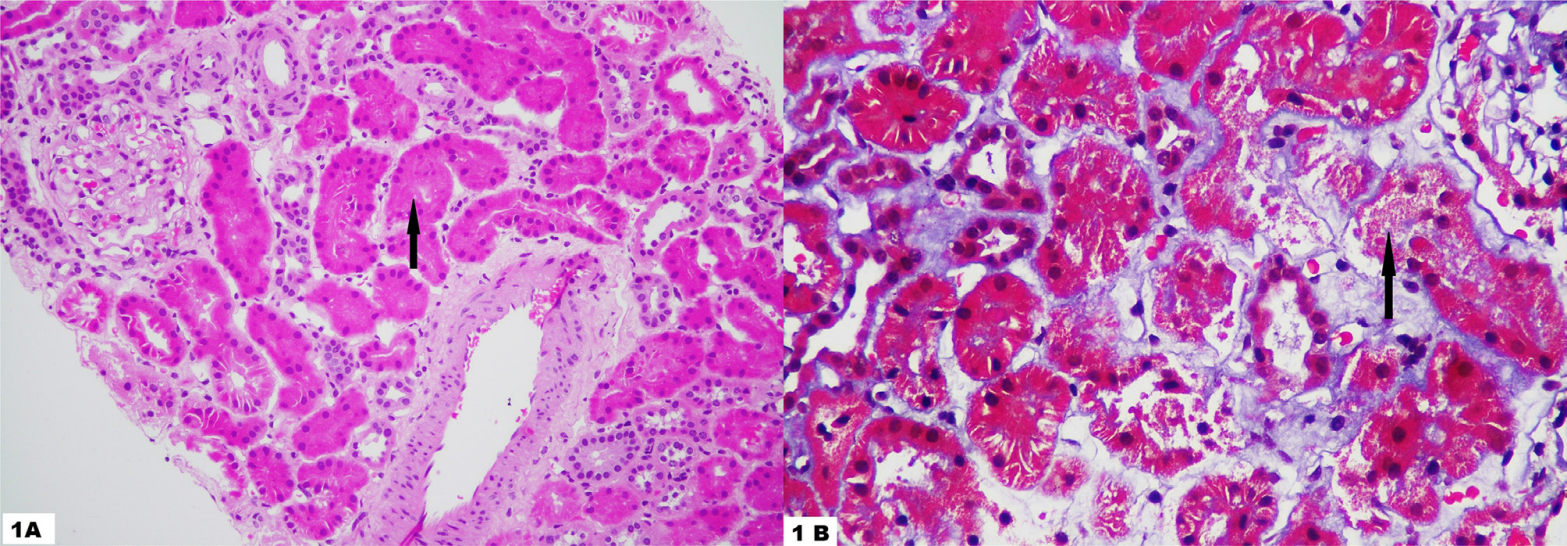
1A: Hematoxylin and eosin staining of renal biopsy core reveals a single normal glomerulus with tubular epithelial cell injury (black arrow) in few tubules without any interstitial fibrosis and tubular atrophy (magnification: 400X); 1B: Masson’s Trichrome staining showing tubules arranged back-to-back with a single tubule (black arrow) showing tubular epithelial cell injury with cell debris in the tubular lumen (Magnification: 400X).

A 26-year-old gentleman, weighing 78 kilograms, presented with history of rhinitis, low grade fever and decreased urine output for 3 days in the nephrology outpatient department in the 3^rd^ week of June. On his master health check-up for employment in January 2023, he had a baseline creatinine of 0.7 mg/dL. On evaluation, he was incidentally found to have creatinine of 2.1 mg/dL. He had a history of a weight gain of 6 kg with easy fatiguability for the last 3 months. He had no significant family history and his drug history included intake of two doses of 500 milligrams paracetamol and 5 mg levocetirizine for rhinitis. He also complained of intermittent myalgia for the last 2 weeks. Clinical examination revealed euvolemia, pulse rate of 58/minute with a blood pressure of 128/68 mm of Hg with urine output of approximately 800 mL/day (less than 0.5 mL/kg/hour). There were no bite marks or discoloration of limbs suggestive of direct muscle injury. In view of his unexplained renal failure, he was admitted on June 26 and a thorough assessment was initiated, which revealed raised serum creatinine of 2.4 mg/dL with normal sodium (136 meq/L), potassium (4.9 meq/L), chloride (102 meq/L), bicarbonate (20 meq/L), and blood sugar levels. Urine examination revealed trace albuminuria with occasional red blood cells/high power field. Hemoglobin was 16.2 grams/dL, white blood cell count of 8070/mm^3^, and platelet count of 208.000/mm^3^. Aspartate transaminase (SGOT) was 69 U/L and alanine transaminase (SGPT) was 51 U/L with normal bilirubin levels. Viral serology workup for hepatitis B surface antigen, anti HCV, and HIV were negative. Ultrasound of the abdomen revealed normal sized kidneys without evidence of obstruction. He had normal serum complement levels, negative urine culture, and negative anti-nuclear antibody levels. Due to his intermittent myalgia, serum total creatine phosphokinase (CPK) level was assessed, which revealed a value of 3869 IU/L with normal CPK-MB fraction. There was no history of direct muscle trauma, crush injury, prior history of recurrent muscle pain or weakness, family history of similar muscle pathology, or inadvertent drug abuse, and he had normal sugar level with normal electrolytes. In view of bradycardia, easy fatiguability, and weight gain, thyroid profile was assessed, which revealed TSH >150 mIU/L, free T3-2.61 picomoles/L (range: 2.1 – 4.4 picomoles/L), and free T4-0.81 nanograms/dL (range: 0.80–2.70 nanograms/dL). Ultrasound revealed heterogeneous, diffusely enlarged thyroid gland with linear echogenic septations suggestive of Hashimoto’s thyroiditis. Serum thyroid peroxidase antibody (anti TPO) level was greater than 1000 IU/mL and Anti Thyroglobulin(anti Tg) levels were 1930 IU/ml. Renal biopsy was done due to unclear cause of renal failure, which revealed 25 glomeruli with normal cellularity and patent capillary loops. Hematoxylin and eosin (Figure 1A) and Masson’s trichrome (Figure 1B) staining revealed sloughing of tubular epithelial cells with cell debris in many tubules, which was indicative of acute tubular injury. Immunofluorescence was negative for all antibodies and immunohistochemistry was positive for myoglobin. Whole exome gene sequencing didn’t reveal any genetic mutation predisposing to rhabdomyolysis. He was diagnosed with acute tubular injury induced by autoimmune thyroiditis and rhabdomyolysis based on raised CPK levels and he was initiated on thyroxine sodium 75 micrograms (mcg) with appropriate hydration for rhabdomyolysis. His creatinine decreased to 1.8 mg/dL after 7 days of admission. The dose of thyroxine was gradually increased by 25 mcg every 2 weeks. On follow-up after 1 month in August 2023, his serum creatinine decreased to 1.2 mg/dL with normal CPK levels (208 IU/L). He is presently on 125 mcg (1.8 mcg/kg) thyroxine sodium with a fortnightly follow up in nephrology department.

## Discussion

Thyroid hormones play a potential role in maintaining renal tubular mass, tubular cell integrity, and tubular re-absorptive capacity^1^. They control the activities of the basolateral Na/K ATPase pump, Na–Pi co-transporter, and apical Na–H exchanger (NHE) on the proximal tubules^1^. Hypothyroidism reduces GFR, impairs RAAS activation, reduces the glomerular filtration surface area, and retards absorption of sodium and phosphorous in the proximal tubules, which result in medullary hypotonicity, fluid retention, reversible creatinine elevation, and consequential hyponatremia^1^.

Thyroid hormones influence the contractility, glycolytic, and oxidative capacities of skeletal muscles by increased expression of T3-mediated proteins like SERCA1a, SERCA2a, GLUT4, and uncoupling protein 3 (UCP3)^2^. Thyroid hormone-mediated expression of MYOD1, T3-mediated myogenic regulatory factor (MRF), and T3-dependent transcription of the gene encoding peroxisome proliferator-activated receptor-γ coactivator-1α are essential for fast fiber phenotype of skeletal muscles, skeletal muscle embryonic development, mitochondrial biogenesis, plasticity, and repair^2^.

Hashimoto’s thyroiditis is characterized by antibody-mediated destruction of thyrocytes with progressive fibrosis resulting in hypothyroidism^3^. It has female preponderance (10:1), the mean age of onset between 30 and 50 years and is most commonly characterized by presence of anti-TPO antibodies followed by anti-Tg antibodies^3^. The clinical features associated with Hashimoto’s thyroiditis include exertional dyspnea, fatigability, exercise intolerance, weight gain, cold dry skin, muscle cramps, bradycardia, slow speech, and ataxia^3^. Hashimoto’s thyroiditis is associated with polyglandular autoimmune syndrome type 2, pernicious anemia, non-steroidal anti-inflammatory drugs (NSAID) intake, coeliac disease, and adrenal insufficiency^3^. Ultrasound usually reveals diffuse enlargement, heterogenous echotexture, hypoechoic micronodules and echogenic linear septations in the thyroid gland^4^. Our patient had elevated TSH with low normal T4, positive anti-TPO, positive anti-TG, and ultrasound neck revealing heterogenous, diffusely enlarged thyroid gland with linear echogenic septations, thereby meeting the criteria for Hashimoto’s thyroiditis.

The acquired causes of rhabdomyolysis include trauma, crush injury, venoms, poisons, toxins, medication such as statins, antihistaminic, anti-psychotics such as aripiprazole and clozapine, tricyclic anti-depressants, and metabolic causes such as diabetes mellitus, hypothyroidism, adrenal insufficiency, and postpartum hypernatremia^5^. Hypothyroidism is a relatively rare cause of rhabdomyolysis, and severe deficiency of thyroid hormones would have compromised structural integrity of skeletal muscles thereby triggering subclinical rhabdomyolysis in our patient following intake of trivial dose of antihistaminic^2,4,6^

The most common renal afflictions associated with autoimmune thyroiditis include focal segmental glomerulosclerosis (20%), membranous nephropathy (20%), chronic glomerulonephritis (15%), IgA nephropathy (15%), minimal change disease (10%), and amyloidosis (5%)^7^. The mechanisms involved in glomerular pathologies are *in-situ* immune complex formation against thyroglobulin at sub-epithelial location, epitope spreading, and cross reactivity of antigens^7^. Our case report is unique in that the renal biopsy showed acute tubular injury. We postulate that megalin expressed in the proximal tubular cells may act as an immunological target for auto antibodies in autoimmune thyroiditis, leading to tubular epithelial cell necrosis^7^. This case report highlights the importance of recognizing hypothyroidism as a reversible factor in the development of acute kidney injury in predisposed individuals^8^ and that appropriate treatment in such clinical scenarios will lead to successful outcomes.

## Data Availability

The data for substantiating the findings of this manuscript are available with the corresponding author and can be made available on request.
