# Supplementary material for: Hashimoto’s thyroiditis masquerading as acute tubular injury and rhabdomyolysis
Source: J Bras Nefrol. 2024 Aug 9;46(3):e20240022. doi: 10.1590/2175-8239-JBN-2024-0022en (PMC11318353; doi:10.1590/2175-8239-JBN-2024-0022en)
Supplement: Translation

# Translation: Tireoidite de Hashimoto disfarçada de lesão tubular aguda e rabdomiólise

**DOI:** 10.1590/2175-8239-JBN-2024-0022pt

**Authors:** Gerry George Mathew, Varadharajan Jayaprakash

**Affiliations:** 1 SRM Medical College Hospital and Research Centre, Department of Nephrology, Chengalpattu, India.

**Keywords:** Doença de Hashimoto, Necrose Tubular Renal Aguda, Rabdomiólise

## Abstract

A tireoidite de Hashimoto que se manifesta como hipotireoidismo tem sido implicada em distúrbios glomerulares devido à formação de autoanticorpos. Apresentamos aqui o caso de um homem de 26 anos de idade, sem quaisquer comorbidades, que apresentou fadiga fácil e ganho de peso por 2 meses. Foi constatado que ele apresentou creatinina de 2,1 mg/dL com histórico de rinite tratada com anti-histamínico três dias antes da visita ao hospital. Ele teve sintomas de mialgia intermitente nas duas semanas anteriores. Na avaliação laboratorial, constatou-se que ele apresentava níveis elevados de CPK, TSH elevado, T4 normal baixo e anticorpos anti-TPO e anti-Tg positivos. A ultrassonografia do pescoço revelou septações ecogênicas lineares na glândula tireoide. A biópsia renal revelou lesão tubular aguda. Foi iniciada uma suplementação adequada de tiroxina e sua creatinina foi reduzida para 1,2 mg/dL após um mês. É importante que os médicos estejam cientes dessa rara apresentação renal na tireoidite de Hashimoto.

## Introdução

Os hormônios tireoidianos são agentes vitais na integridade estrutural e funcional dos rins e dos músculos esqueléticos^1,2^. A tireoidite de Hashimoto geralmente se manifesta como hipotireoidismo devido à destruição dos tireócitos com a presença de autoanticorpos e apresenta características ultrassonográficas^3,4^. A rabdomiólise tem sido pouco documentada na literatura médica em casos de hipotireoidismo grave^5,6^. A tireoidite autoimune tem sido implicada em diversas patologias glomerulares devido ao fenômeno da formação de autoanticorpos^7^. Neste artigo, relatamos uma manifestação renal rara de tireoidite autoimune, que tem uma implicação importante em termos de diagnóstico e manejo.

## Descrição do Caso

Um homem de 26 anos, pesando 78 kg, apresentou-se no ambulatório de nefrologia na terceira semana de junho com histórico de rinite, febre baixa e diminuição do débito urinário durante três dias. Em seu exame médico geral realizado para o trabalho em janeiro de 2023, ele apresentou uma creatinina basal de 0,7 mg/dL. Na avaliação, descobriu-se incidentalmente que ele apresentava creatinina de 2,1 mg/dL. Ele tinha um histórico de ganho de peso de 6 kg com fadiga fácil nos últimos 3 meses. O paciente não tinha histórico familiar significativo e seu histórico de medicamentos incluía a ingestão de duas doses de 500 miligramas de paracetamol e 5 mg de levocetirizina para rinite. Ele também se queixou de mialgia intermitente nas últimas duas semanas. O exame clínico revelou euvolemia, frequência de pulso de 58/minuto com pressão arterial de 128/68 mmHg e débito urinário de aproximadamente 800 mL/dia (menos de 0,5 mL/kg/hora). Não havia marcas de mordidas ou descoloração de membros que sugerissem lesão muscular direta. Em virtude de sua insuficiência renal inexplicável, ele foi internado em 26 de junho e iniciou-se uma avaliação completa, a qual revelou creatinina sérica elevada de 2,4 mg/dL com níveis normais de sódio (136 meq/L), potássio (4,9 meq/L), cloreto (102 meq/L), bicarbonato (20 meq/L) e glicemia. O exame de urina revelou traços de albuminúria com presença ocasional de hemácias/campo de grande aumento. A hemoglobina foi de 16,2 gramas/dL, a contagem de leucócitos foi de 8070/mm^3^, e a contagem de plaquetas foi de 208.000/mm^3^. A aspartato aminotransferase (AST) foi de 69 U/L e a alanina aminotransferase (ALT) foi de 51 U/L com níveis normais de bilirrubina. Os exames de sorologia viral para antígeno de superfície da hepatite B, anti-HCV e HIV foram negativos. A ultrassonografia abdominal revelou rins de tamanho normal sem evidência de obstrução. Ele apresentou níveis normais de complemento sérico, cultura de urina negativa e níveis negativos de anticorpos antinucleares. Devido à sua mialgia intermitente, o nível sérico total de creatina fosfoquinase (CPK) foi avaliado, revelando um valor de 3869 UI/L com fração CPK-MB normal. Não houve histórico de trauma muscular direto, lesão por esmagamento, histórico prévio de dor ou fraqueza muscular recorrente, histórico familiar de patologia muscular semelhante ou abuso inadvertido de drogas, e os níveis de glicose e eletrólitos estavam normais. Diante da bradicardia, fadiga fácil e ganho de peso, foi avaliado o perfil tireoidiano, que revelou TSH >150 mIU/L, T3 livre de 2,61 picomoles/L (variação: 2,1 – 4,4 picomoles/L) e T4 livre de 0,81 nanogramas/dL (variação: 0,80–2,70 nanogramas/dL). A ultrassonografia revelou glândula tireoide heterogênea, difusamente aumentada, com septações ecogênicas lineares sugestivas de tireoidite de Hashimoto. O nível sérico de anticorpo contra a peroxidase tireoidiana (anti-TPO) foi superior a 1.000 UI/mL e os níveis de anti-tireoglobulina (anti-Tg) foram de 1.930 UI/ml. Realizou-se biópsia renal devido à causa incerta da insuficiência renal, a qual revelou 25 glomérulos com celularidade normal e alças capilares pérvias. A coloração com hematoxilina e eosina (Figura 1A) e tricrômico de Masson (Figura 1B) revelaram descamação de células epiteliais tubulares com detritos celulares em muitos túbulos, o que foi indicativo de lesão tubular aguda. A imunofluorescência foi negativa para todos os anticorpos e a imuno-histoquímica foi positiva para mioglobina. O sequenciamento do exoma completo não revelou nenhuma mutação genética que predispusesse à rabdomiólise. Ele foi diagnosticado com lesão tubular aguda induzida por tireoidite autoimune e rabdomiólise com base nos níveis elevados de CPK e foi iniciado tratamento com tiroxina sódica 75 microgramas (mcg) com hidratação adequada para rabdomiólise. Sua creatinina diminuiu para 1,8 mg/dL após 7 dias de internação. A dose de tiroxina foi aumentada gradualmente em 25 mcg a cada duas semanas. No acompanhamento após um mês, em agosto de 2023, sua creatinina sérica havia diminuído para 1,2 mg/dL com níveis normais de CPK (208 UI/L). Atualmente, ele está em tratamento com 125 mcg (1,8 mcg/kg) de tiroxina sódica, com acompanhamento quinzenal no departamento de nefrologia.

**Figura 1:** 1A: A coloração com hematoxilina e eosina do núcleo da biópsia renal revela um único glomérulo normal com lesão de células epiteliais tubulares (seta preta) em poucos túbulos sem qualquer fibrose intersticial e atrofia tubular (ampliação: 400X); 1B: Coloração com Tricrômico de Masson mostrando túbulos dispostos lado a lado com um único túbulo (seta preta) mostrando lesão de células epiteliais tubulares com detritos celulares no lúmen tubular (Ampliação: 400X).

## Discussão

Os hormônios tireoidianos desempenham um papel potencial na manutenção da massa tubular renal, da integridade das células tubulares e da capacidade de reabsorção tubular^1^. Eles controlam as atividades da bomba Na/K ATPase basolateral, do co-transportador Na-Pi e do trocador Na-H (NHE) apical nos túbulos proximais^1^. O hipotireoidismo reduz a TFG, prejudica a ativação do SRAA, reduz a área de superfície de filtração glomerular e retarda a absorção de sódio e fósforo nos túbulos proximais, o que resulta em hipotonicidade medular, retenção de fluidos, elevação reversível da creatinina e consequente hiponatremia^1^.

Os hormônios tireoidianos influenciam a contratilidade, as capacidades glicolítica e oxidativa dos músculos esqueléticos por meio do aumento da expressão de proteínas mediadas por T3, como SERCA1a, SERCA2a, GLUT4 e proteína desacopladora 3 (UCP3)^2^. A expressão de MYOD1 mediada pelo hormônio tireoidiano, o fator regulador miogênico (MRF) mediado por T3 e a transcrição dependente de T3 do gene que codifica o coativador-1α do receptor γ ativado por proliferador de peroxissoma são essenciais para o fenótipo de fibra rápida dos músculos esqueléticos, desenvolvimento embrionário do músculo esquelético, biogênese mitocondrial, plasticidade e reparo^2^.

A tireoidite de Hashimoto é caracterizada pela destruição dos tireócitos mediada por anticorpos com fibrose progressiva, resultando em hipotireoidismo^3^. Apresenta preponderância feminina (10:1), idade média de início entre 30 e 50 anos e é mais comumente caracterizada pela presença de anticorpos anti-TPO seguidos de anticorpos anti-Tg^3^. As características clínicas associadas à tireoidite de Hashimoto incluem dispneia aos esforços, fatigabilidade, intolerância ao exercício, ganho de peso, pele seca e fria, cãibras musculares, bradicardia, fala lenta e ataxia^3^. A tireoidite de Hashimoto está associada à síndrome autoimune poliglandular tipo 2, anemia perniciosa, ingestão de anti-inflamatórios não esteroidais (AINEs), doença celíaca e insuficiência adrenal^3^. A ultrassonografia geralmente revela aumento difuso, ecotextura heterogênea, micronódulos hipoecoicos e septações ecogênicas lineares na glândula tireoide^4^. Nosso paciente apresentou TSH elevado com T4 normal baixo, anti-TPO positivo, anti-TG positivo e ultrassom do pescoço revelando glândula tireoide heterogênea e difusamente aumentada com septações ecogênicas lineares, atendendo, assim, aos critérios para tireoidite de Hashimoto.

As causas adquiridas de rabdomiólise incluem trauma, lesão por esmagamento, venenos, substâncias tóxicas, medicamentos como estatinas, anti-histamínicos, antipsicóticos como aripiprazol e clozapina, antidepressivos tricíclicos e causas metabólicas como diabetes mellitus, hipotireoidismo, insuficiência adrenal e hipernatremia pós-parto^5^. O hipotireoidismo é uma causa relativamente rara de rabdomiólise, e a deficiência grave de hormônios tireoidianos teria comprometido a integridade estrutural dos músculos esqueléticos, desencadeando assim a rabdomiólise subclínica em nosso paciente após a ingestão de uma dose trivial de anti-histamínico^2,4,6^

As afecções renais mais comuns associadas à tireoidite autoimune incluem glomeruloesclerose segmentar focal (20%), nefropatia membranosa (20%), glomerulonefrite crônica (15%), nefropatia por IgA (15%), doença de lesão mínima (10%) e amiloidose (5%)^7^. Os mecanismos envolvidos nas patologias glomerulares são a formação *in situ* de complexos imunes contra a tireoglobulina na localização subepitelial, a disseminação de epítopos e a reatividade cruzada de antígenos^7^. Nosso relato de caso é único, visto que a biópsia renal mostrou lesão tubular aguda. Postulamos que a megalina expressa nas células tubulares proximais pode atuar como um alvo imunológico para autoanticorpos na tireoidite autoimune, levando à necrose das células epiteliais tubulares^7^. Esse relato de caso destaca a importância de reconhecer o hipotireoidismo como um fator reversível no desenvolvimento de injúria renal aguda em indivíduos predispostos^8^ e que o tratamento adequado em tais cenários clínicos levará a desfechos bem-sucedidos.

## Data Availability

Os dados para fundamentar os achados deste manuscrito estão disponíveis com o autor correspondente e podem ser disponibilizados mediante solicitação.
